# Sensor-Based Monitoring of Fire Precursors in Timber Wall and Ceiling Assemblies: Research Towards Smarter Embedded Detection Systems

**DOI:** 10.3390/s25154730

**Published:** 2025-07-31

**Authors:** Kristian Prokupek, Chandana Ravikumar, Jan Vcelak

**Affiliations:** University Centre for Energy Efficient Buildings, Czech Technical University in Prague, 273 43 Bustehrad, Czech Republic; ravikcha@partner.cvut.cz (C.R.); jan.vcelak@cvut.cz (J.V.)

**Keywords:** smart building systems, early warning systems, fire safety engineering for timber constructions, pre-ignition monitoring, smoke sensors, integrated diagnostics, in situ structural monitoring

## Abstract

**Highlights:**

A large-scale fire experiment was conducted using a prototype timber building structure. We focused on gases produced in the early stages of a fire and the use of multiple types of sensors for possible detection leading to the development of a more advanced sensor.

**What are the main findings?**

- Specific gas portfolio.
- Earlier fire detection than conventional smoke sensors.

**What is the implication of the main finding?**

- Possibility to improve fire protection of timber buildings.
- Design a multisensory detector for life/property savings.

**Abstract:**

The movement towards low-emission and sustainable building practices has driven increased use of natural, carbon-based materials such as wood. While these materials offer significant environmental advantages, their inherent flammability introduces new challenges for timber building safety. Despite advancements in fire protection standards and building regulations, the risk of fire incidents—whether from technical failure, human error, or intentional acts—remains. The rapid detection of fire onset is crucial for safeguarding human life, animal welfare, and valuable assets. This study investigates the potential of monitoring fire precursor gases emitted inside building structures during pre-ignition and early combustion stages. The research also examines the sensitivity and effectiveness of commercial smoke detectors compared with custom sensor arrays in detecting these emissions. A representative structural sample was constructed and subjected to a controlled fire scenario in a laboratory setting, providing insights into the integration of gas sensing technologies for enhanced fire resilience in sustainable building systems.

## 1. Introduction

Advances in sensor technology and the Internet of Things (IoT) have opened up new possibilities for fire detection in buildings. Modern fire detection systems increasingly use multi-sensor detectors that combine smoke, heat, and gas sensing with algorithmic analysis to distinguish real fires from false alarms. At the same time, there is a growing trend of false alarms caused (48.2% in year 2024 [1]) by EPS (Electronic Protective Systems)/sensors. This fact needs to be reflected in new solutions.

Traditional fire detection in buildings relies on commercial smoke detectors. They are based on optical, temperature (heat), or particle-ionization principles. Gas sensors are not usually used for smoke or fire detection; they are primarily used for gas leaking detection or the prevention of explosion or poisoning. Specifically, gas sensors (for CO, CO_2_, VOC, etc.) can be integrated into our experiment to detect the unique gaseous signatures before and during fires. CO sensors are already used in home/garage alarms to detect smoldering fires or toxic gas presence. CO sensors react more quickly than photoelectric smoke detectors alone. Research since 2017 [2] has accelerated in developing specialized sensors for early fire gases and wireless sensor networks for buildings. For example, low-power metal-oxide semiconductor (MOS) sensors can continuously monitor for slight increases in CO or H_2_ [3], and AI algorithms can analyze patterns from multiple sensors to decide alarm condition.

This study also uses smoke and gas sensors to address a critical need in fire safety by detecting gas emissions from individual components of composite timber assemblies during fire exposure. We hypothesize that each structural layer (named in Figure 4) produces a distinct gas signature under intense heat, and that detecting these unique chemical precursors can guide the development of advanced fire sensors that are responsive to early material-specific emissions rather than generic combustion products. It should be noted that the introduced word “precursor” is used in the sense of an indicator of a possible situation, i.e., in the case of this experiment it is a precursor to the possible occurrence of a fire. A precursor is a mixture of gases and VOCs given by its composition and heat conditions.

Fires can expand at an exponential rate; experiments have shown fire size doubling in times as short as 20–30 s under certain conditions [4,5], and flashover—the point where all combustible materials ignite simultaneously—can occur within minutes. This means a fire detected in its incipient stage provides a window of opportunity for rapid intervention that would not exist if the fire is only discovered later. As a fire grows, it generates exponentially more heat and smoke, which not only increases property damage but also reduces human survivability. Thick smoke and toxic gases can render occupants’ unconscious or unable to escape if too much time passes. Indeed, roughly half of home fire deaths occur between 11:00 p.m. and 7:00 a.m.—when people tend to be asleep—precisely because the fire can smolder and spread unnoticed for longer before anyone is alerted [6].

**Figure 1:**
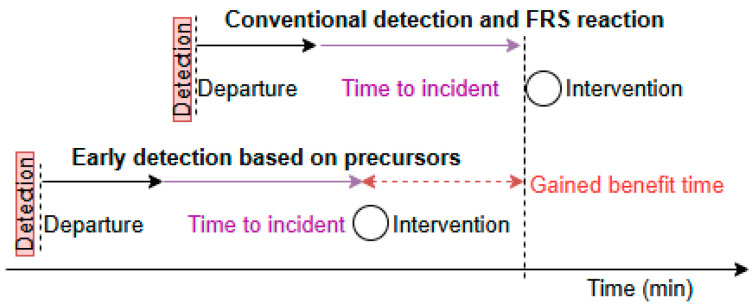
Illustration of the time benefit due to early detection proposed by the authors.

When a fire is detected, firefighters face unavoidable delays in physically reaching the scene. The average time to arrival at the scene of an incident is 7.5 min in Czechia [1]. The arrival time depends on many factors, let us assume we cannot influence it (i.e., weather, traffic, route). Although 70% of nearest FRS (“fire rescue service”) units are located within 5 km of the site of emergency. The Figure 1 illustrates the timeline between the early detection proposed in this study and the arrival of fire rescue services.

While it is challenging to eliminate all response delays, earlier detection systems can provide valuable lead time. Early detection allows them to give early alerts enabling occupants to evacuate before conditions become life-threatening. It also helps in timely suppression, if the automatic suppression systems are activated upon early detection, it can control or extinguish fires before they spread. Also, early information allows the fire services to assess the situation accurately and allocate appropriate resources swiftly. These scenarios underline the fact that early fire detection literally creates time—a few extra minutes—that can mean the difference between a minor incident and a major tragedy.

## 2. Existing Works

In the Czech Republic, for example, roughly 15% of newly built houses are timber-framed [1,4]. Europe overall still has a relatively small proportion of wood-based buildings—in the order of <10% of the building stock [3,5]—yet some regions far exceed this. Sweden leads in timber construction, with nearly 90% of new single-family homes constructed primarily from wood [7]. Other countries show more moderate but significant adoption: Austria sees about 33% of new houses built with timber structures, Germany around 25–30% [7], and the United Kingdom has approximately 20–28% [4,6]. In Czechia, current legislation does not allow the timber construction of multi-story buildings, but other EU countries are further along in these constructions, and it is therefore expected that legislation and standards could be amended in the future. These statistics show that a substantial share of buildings in many regions are wood-based, highlighting the critical need for prompt fire detection systems in these buildings.

Spruce is the most common wood construction material in Czechia. Recognizing these differences is crucial for understanding wood behavior during fires, providing a scientific basis for fire safety design. Researchers have conducted numerous experimental studies and mechanistic investigations into the combustion characteristics of timber.

**Figure 2:**
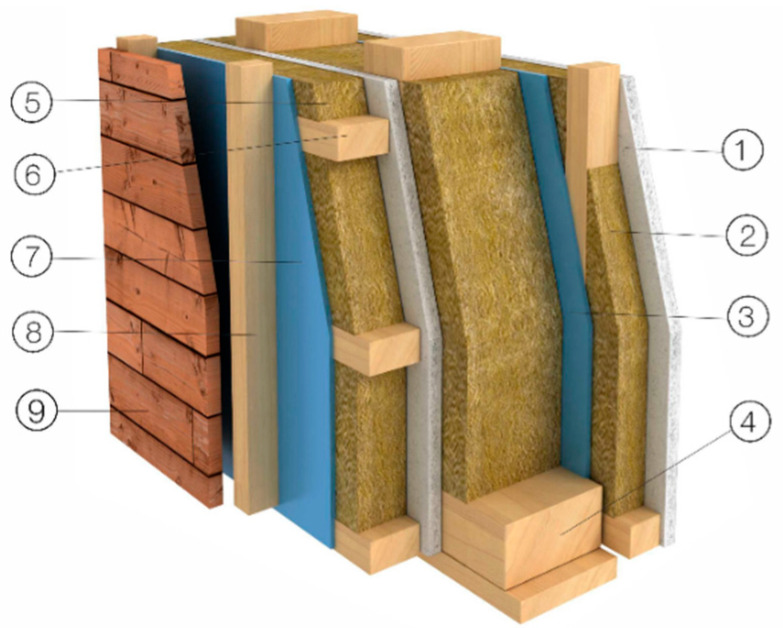
Scheme of typical timber wall with wooden cladding [8].

To ensure relevance and applicability, our experimental setup was based on the standardized material compositions used by Czechia’s leading manufacturer [8], who is also known for high-volume production of timber-frame houses and single-story buildings. Figure 2 illustrates a typical wall assembly used in such constructions, consisting of multiple functional layers. For the purposes of this study, we focused on the inner layers most representative of fire-prone components: (1) a 15 mm gypsum fiber board (“Fermacell”), (2) a wooden grate filled with thermal insulation, (3) a vapor barrier membrane, (4) a structural timber frame filled with mineral wool insulation, (5) another layer of gypsum fiber board, (6) a second wooden grate with thermal insulation, (7) a diffusion open-foil membrane, (8) a ventilated cavity formed by an external wooden grate, and (9) outer timber cladding. This layered configuration reflects timber construction practices and provides a representative test sample composition for laboratory specimen construction.

Oriented Strand Board (OSB) emits water vapor, VOCs (formaldehyde, acetic acid), CO, and CO_2_ during pre-ignition, with adhesives releasing toxic gases like formaldehyde, ammonia, and isocyanates; combustion produces high levels of CO, HCN (up to 200 ppm), aldehydes, PAHs, and dense toxic smoke—making OSB up to 20× more hazardous than solid wood in under-ventilated fires [9]. Plasterboard, in contrast, primarily emits steam (~1.7 kg/m^2^) with negligible VOCs and remains inert post-ignition, producing minimal CO/CO_2_ and no hazardous gases like HCN or NO_x_. Polyethylene vapor barriers release flammable hydrocarbons (methane, ethylene) and toxic oxygenates (formaldehyde, acrolein) during pyrolysis, followed by large CO_2_/CO outputs, black smoke, and PAHs upon combustion, posing high smoke toxicity despite lacking nitrogen or halogens [10]. Mineral wool insulation emits trace gases (formaldehyde, HCN, CO) only from its small binder content, with overall emissions remaining low; the inorganic core stays inert up to ~1300 °C, making it a relatively fire-safe material [11].

Table 1 summarizes the ignition properties, gases released during combustion phases, and typical quantities for key timber components and associated construction materials.

**Figure 3:**
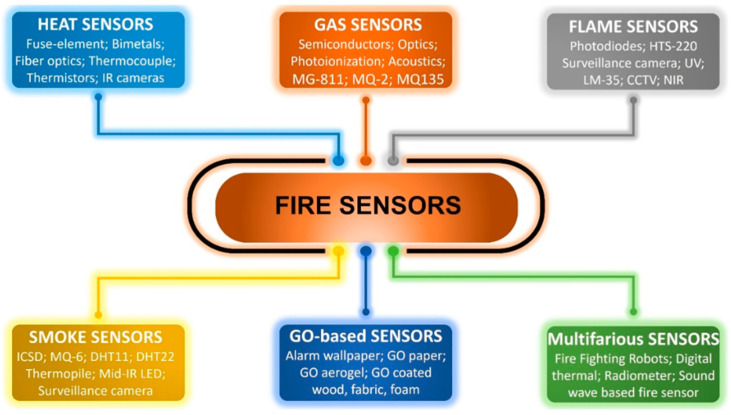
A summary of current fire sensing technologies.

However, over time, fire detection technologies have significantly evolved, encompassing various sensing methods such as heat, gas, flame, smoke, and emerging graphene oxide (GO)-based sensors. Gas sensors, especially those based on semiconductor metal oxides (MOS), are valued for their sensitivity, compactness, and cost-effectiveness, though stability issues persist. Research is also exploring carbon nanotube-based gas sensors for fire detection. All of these current sensing technologies are illustrated in Figure 3.

Current studies often rely on standard fire exposure tests (e.g., ISO 834-1 [16], ASTM E119) [15], which may not accurately represent real-world fire scenarios, particularly in large or complex timber buildings, they often emphasize primary combustion products like carbon monoxide (CO) and carbon dioxide (CO_2_), while overlooking other hazardous compounds such as acrolein, formaldehyde, and hydrogen cyanide, particularly under varying ventilation conditions.

There is a notable scarcity of full-scale fire tests that replicate realistic building scenarios, including the interaction of multiple materials and varying fire loads, which limits the applicability of findings to actual fire events. Additionally, the impact of material aging on fire behavior and toxic emissions remains underexplored, especially materials like polyethylene and mineral wool that may degrade over time. While computational models like the Fire Dynamics Simulator (FDS) are employed to predict fire behavior [17,18], there is a pressing need for more extensive validation against experimental data to ensure their accuracy in forecasting toxic emissions and fire spread.

To address these gaps, this study attempted to conduct fire experiments that mimic real building configurations and can provide valuable insights into the interaction of different materials and the resulting gas emissions, while also comparing the analyses by gas sensors and smoke (fire) sensors.

## 3. Materials and Methods

**Figure 4:**
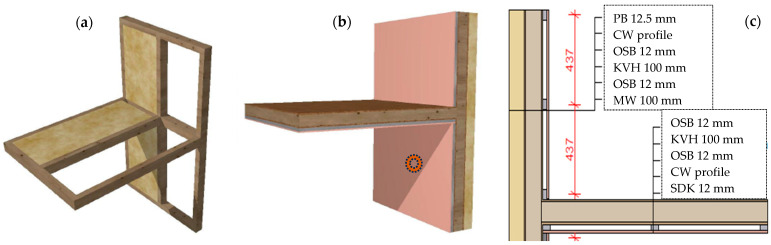
Primary idea of test sample construction in views: (**a**) Two hollows constructed with/without insulation. (**b**) Completed test specimen (wall and ceiling timber construction). (**c**) Description of specimen wall composition.

The test specimen was designed in collaboration with experts in fire testing, building construction, and sensor systems. The goal was to create a representative wall and ceiling assembly using standard timber construction materials. The design is based on a local manufacturer’s system (Figure 2) but we simplified the composition while keeping the original materials (Figure 4). Fire is expected to start inside the room. Also, we used a simplified version of the external facade and insulation, as fire spread to the outside within an hour is unlikely due to heavy MV insulation and based on fire testing experts’ knowledge. However, this will still be monitored with cameras. The construction is made to include insulated and uninsulated timber panels with two separate cavities, as shown in Figure 4, allowing one to test both fire behaviors.

The wall structure, shown in Figure 4c, includes mineral wool insulation (Knauf, Czech Republic), OSB (Kronospan, Jihlava, Czech Republic), a frame made from spruce timbers (10 × 10 cm, Wood Store s.r.o., Czech Republic), metal profiles (Knauf, Czech Republic), and a single layer of plasterboard (PB) (Knauf, Czech Republic). A vapor-barrier and vapor-permeable membrane (VM) (Den Braven, Czech Republic) was added in the usual position and was the only plastic material used. To reduce fire load, we avoided polystyrene insulation and used mineral wool with oriented fibers for the facade. The specimen includes a two-story wall and one ceiling section to reflect typical building conditions, with the total height adapted to fit within the fire chamber.

The configuration was designed to create different fire spread conditions, with the uninsulated cavity expected to allow faster fire development through the thinner wall section. The central beam framework provided clear separation between the two cavities, enabling two fire exposure scenarios to be tested within a single specimen.

The total experiment duration was set to 60 min with an assessment checkpoint at 30 min, where the construction and integrity were considered concerning the continuation of the experiments. This period did not include the initial burner ignition phase. The test period includes transition to full heat output, and stable exposure according to the intended fire profile. The decision aligned with the common fire resistance classification EI60 and followed the relevant standards EN 13501-2 [19] and EN 13238 [20].

The test specimen (Figure 4) was constructed with two sets of vertically arranged cavities, visible from the front elevation (Figure 4a). The cavities on the left were filled with mineral wool insulation (MV) to match the depth of the timber structural members (KVH–timber prism), while the cavities on the right were intentionally left uninsulated.

The ignition source (gas burners in the burner chamber) was located on the first floor. The flame was directed towards the center of the lower floor wall (marked in Figure 4b). This location was chosen to represent a typical electrical appliance plug connection, as a possible common location for heating or fire in the event of a fault.

The methodology involved assembling the specimen, integrating the sensor systems, placing the setup in the fire laboratory, and conducting the fire exposure test for a defined duration of 60 min. Conduct of experiment is more described in Appendix A.

While advanced gas analysis methods such as FTIR (Fourier Transform Infrared Spectroscopy) or GC (Gas Chromatography) were not available, the study demonstrates a resource-efficient approach to monitoring combustion gases. Commercially available gas sensors Fermion (DFRobot Co., Ltd., Shanghai, China), Moisture guard [21] and IAQ (Indoor Air Quality) by Senzomatic (MoistureGuard s.r.o., Prague, Czechia) were selected to assess the real-time trends in the gas concentration, despite known limitations in selectivity and cross-sensitivity. This choice reflects a deliberate and justified trade-off, ensuring continuous data collection while maintaining experimental simplicity and robustness.

We also evaluated the possibility of post-analysis using gas sampling bags (i.e., Tedlar air sampling bags (Tedlar®, Sigma-Aldrich); however, concerns over VOC degradation and strict timing requirements to transfer the samples to the gas analytical laboratory made this approach impractical.

Supporting temperature monitoring was achieved using strategically placed pyrometers (Optris), capable of accurate spot measurements from a 1 m distance. These sensors, connected via USB and operated with custom-developed CompactConnect (software version 1.10.11), enabled reliable surface temperature tracking without direct exposure to flames. Together, these methods form a coherent, well-justified experimental framework that offers novel insights into fire behavior in timber constructions under constrained conditions—highlighting both practical innovation and methodological soundness.

Given the department’s expertise in smart building technologies, the experiment leveraged in-house sensor systems to enhance data quality and relevance. MoistureGuard sensors —part of the Senzomatic system [21] and validated in over 1000 installations—were employed to monitor temperature and humidity. These sensors are commonly used in timber buildings for long-term monitoring, making their application in this fire test both practical and directly transferable to real building scenarios. All sensor data were automatically collected and stored in a cloud database accessed by the authors of [21] and the research institute, ensuring secure and centralized data access.

To broaden environmental monitoring, indoor air quality (IAQ) sensors were also integrated into the specimen. Although typically used in occupied environments, their internal capabilities enabled the measurement of temperature, humidity, particulate matter (PM10 and PM2.5), and volatile organic compounds (VOCs) with more VOC-integrated sensors on board. This provided redundancy across key parameters, increasing data reliability in case of local sensor failure. The IAQ sensors [22] (CVUT UCEEB, Bustehrad, Czechia) were connected via a serial bus (RS-485 using the Modbus RTU protocol) to a central computer running custom data acquisition software developed in National Instruments LabVIEW (version 2023 64-bit).

Large-scale thermal surveillance was ensured using an VarioCam HD (Infratec GmbH, Germany), passive infrared (PIR) thermal camera operating in continuous video mode. It was positioned 3 m from the test facade, where it monitored burning through the wall and facade, and monitored possible thermal hotspots while being shielded from direct heat in the fire laboratory premises.

An additional Raspberry Pi platform (Raspberry Pi Holdings plc, Cambridge, UK) with two cameras, a “Wide Raspberry PI Camera 3” (Raspberry Pi Holdings plc, Cambridge, UK) and an “IR Heimann 80 × 64 pixel camera” (Heimann Sensor GmbH, Dresden, Germany), was placed near the fire experiment to capture an angled view of the specimen.

Personal visual documentation was also possible during the experiment, either from safe observation points or through the chamber’s protective glass. This integrated sensor approach demonstrates a well-structured monitoring strategy suited for the real-time evaluation of fire behavior in timber constructions; all the sensors are listed in Table 2 below.

The Fire Laboratory Control System (FLCS) is a custom-built platform developed for managing and recording fire experiments, including control of the exhaust hood and monitoring of combustion emissions. It operates on a proprietary operating system (OS) and uses proprietary software (SW). The FLCS is equipped with hard-wired sensors, including gas analyzers for CO, CO_2_, and O_2_ in the exhaust stack, and an input unit supporting dozens of thermocouples. In this experiment, it was primarily used to monitor temperatures on the test specimen and burner chamber.

For all additional sensors and measurement systems, departmental equipment and computers were used. To analyze gas concentrations and evaluate the performance of smoke and fire detectors, a CompactRIO by National Instruments (“NI” a part of Emerson Electric, Austin, Texas, USA) was employed as DAQ. This module was connected via USB to a PC running LabVIEW on Windows 11. This setup allowed real-time acquisition and comparison of gas sensor data and detector responses. All measurement systems used in laboratory experiments are briefly highlighted in Table 2 below.

## 4. Experiment Implementation

This section describes the execution of the fire experiment and its implementation. The test specimen was assembled in the assembly hall, where sensors, wiring, and auxiliary connections were integrated during the initial construction phase—accounting for approximately half of the total assembly time. Once the structural elements were complete, the specimen was finished with mineral wool insulation and plasterboard. All sensors were verified using appropriate tools such as multimeters, thermocouple testers, and PCs to ensure correct operation.

The completed specimen was transported to the fire laboratory. After removing the transport base, the entire first floor, including the ceiling, was positioned directly onto the burner chamber at the same elevation. From this point, the test specimen stood unsupported, with its ceiling resting on the burner chamber’s upper surface. All subsystems (Table 2) were then connected and tested using their respective software. Following successful verification, the fire experiment was initiated: the four burners in the chamber were ignited (stand-by). After increasing burner power, the experiment proceeded for 60 min, during which data were continuously recorded by two computers and the FLCS. The test was observed through cameras and a laboratory viewing window. When self-sustained combustion within the structure intensified (as seen in the Figures below), the burner output power was reduced. Live measurements were monitored throughout, and any anomalies were logged by text or photograph for subsequent analysis. Immediately after the test concluded, all systems were rapidly disconnected to allow prompt extinguishing of the specimen. Therefore, not all measurement systems were stopped correctly. Hence, the measured data is trimmed from the end of the experiment.

**Figure 5:**
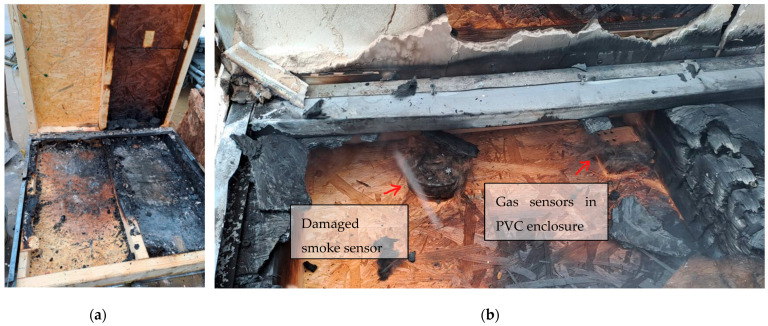
(**a**) Test sample after experiment, partially disassembled. (**b**) Damaged sensors near wall and ceiling joint.

The main sensors relevant to this research—the smoke and gas sensors—were placed on the lower floor, under the upper beam (Figure 4a and Figure 5b). We chose this location because smoke and hot gases would rise in this direction and at the same time we could not expose the sensors to direct fire, which would occur first in the central area.

## 5. Data Processing

The last phase of the experiment focused on analyzing experimental data. During the experiment, we took notes, photographs, videos, time records, and data from several sources, namely subsystems (Figure 2).

The measured data that we entered into Microsoft Excel on individual pages were crucial to the assessment. We chose Excel for its compatibility with CSV and Labview data formats. We synchronized and re-sampled the data series used for up to 3 s for optimal processing. Research data were reduced to the observed time window (0.60 min). We also searched for extreme peaks, local maximums, and minimums and tried to identify them based on these measurements (photos, notes, temperatures).

**Figure 6:**
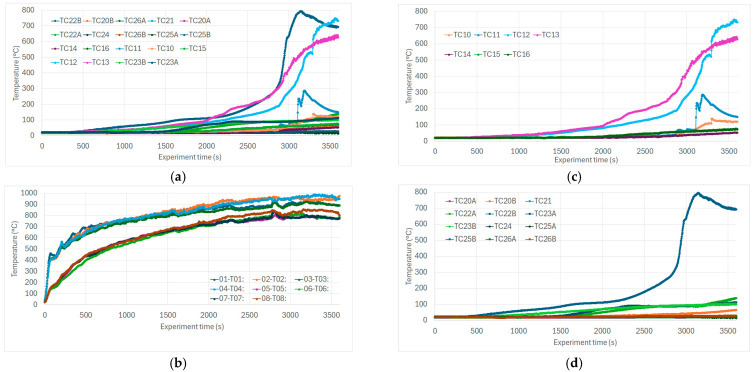
(**a**) All temperature trends from thermocouples on the test specimen. (**b**) Burner temperatures. (**c**) Temperatures in the specimen in the hollow section showing progressive burn-through. (**d**) Temperatures in the specimen in the insulated section. Note: Due to the quantity of presented curves, the colors/curves in (**a**–**d**) do not correspond. Figure 6 provides a clear view of the temperature range within which the experiment was conducted. The observed events correlate with the sharp change in temperatures.

**Figure 7:**
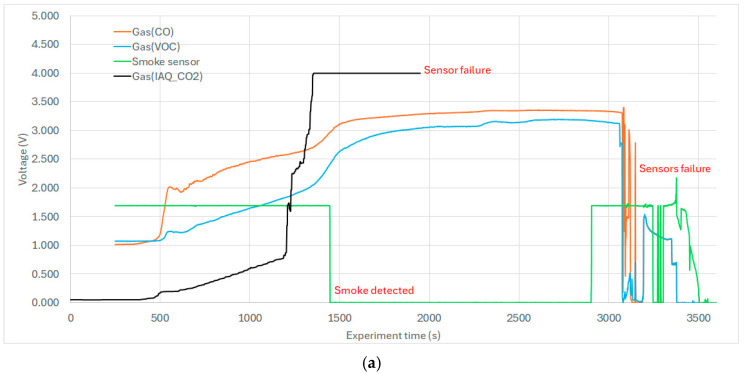

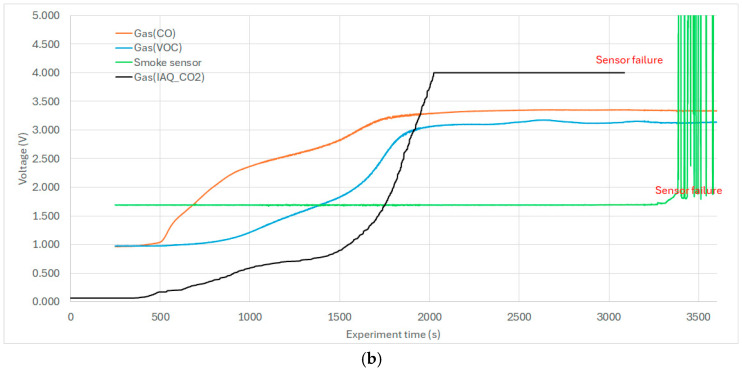
(**a**) Gas, smoke, and CO_2_ sensors in uninsulated hollow section. (**b**) Gas, smoke, and CO_2_ sensors in mineral wool-insulated hollow section.

**Figure 8:**
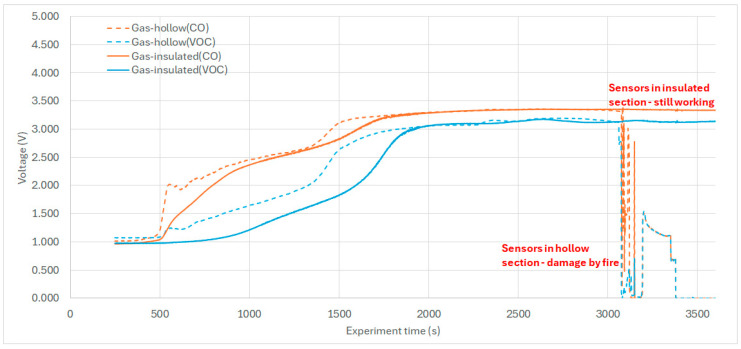
VOC and CO gas sensor response during the experiment.

There was no need to further filter the data series, as we presented such a time course in Figure 6, Figure 7 and Figure 8. Graphs were created to visualize key variables such as temperature and gas concentrations (CO, VOC, CO_2_ sensors) either individually or in combination. Sensor positions and variable labels were included to facilitate the interpretation.

## 6. Results

This section summarizes the course of the experiment, the subsequent data processing and evaluation, and the assessment of the original hypothesis.

### 6.1. Visual Evaluation

The post-experiment assessment was conducted after the specimen had cooled and during its disassembly in the following hours. Burned sections were documented photographically (one of examples in Figure 5). The timber beam structure remained visually stable, though localized surface charring was observed in several areas (Figure 5a). During inspection, several sensors were confirmed to have failed due to fire exposure (Figure 5b), which correlated with abrupt drops or irregularities in the recorded data. PVC cable (industry standard CAT5E 8 × 0.25 mm^2^) damage was also observed (Figure 5)—burned insulation in some areas led to exposed copper conductors and short circuits that explain signal fluctuations in specific sensors (Figure 7 and Figure 8).

### 6.2. Evaluation of the Thermometer Data

Temperature data were analyzed by generating time-series charts, interpreted alongside experiment notes and sensor placement details. Due to the large number of thermocouples embedded in various parts of the structure, a selective display of specific sensors was necessary for clarity or individual explanation.

The initial analysis focused on the hollow section, where a temperature surge (burning thru structure and accelerated self-burning) occurred between T = 2600–2800 s, indicating structural ignition and burn-through of specimen (Figure 6a). This behavior matched expectations for the uninsulated cavity, as also evident in the post-test specimen (Figure 6b).

Burner temperatures are not significant for this research paper, and they were plotted separately (Figure 6c). At around T = 2800 s, a rise in temperature prompted a controlled reduction in burner output, as combustion within the test structure had already begun. This response helped maintain the experiment within the intended conditions and explains the events that occurred at specified times.

Additional graphs focused on specific sections of the specimen. One showed the complete burnout of the uninsulated cavity (Figure 6d). Another highlighted the burn-through into the insulated section using thermocouple pairs placed before and after the insulation layer. Dashed lines represent sensors positioned closer to the burner.

### 6.3. Evaluation of Gas and Smoke Sensor Data

To interpret the gas and smoke sensor data (Figure 7), it was essential to correlate their outputs with temperature trends (Figure 6). In the hollow section below the ceiling joint, the sensors recorded an early rise in volatile organic compounds (VOCs) and carbon monoxide (CO), indicating material heating. This was followed by an increase in carbon dioxide (CO_2_), a byproduct of combustion (Figure 7a). At approximately T = 17 min, structural burn-through occurred, allowing combustion gases to enter the cavity. By T = 21 min, the commercial smoke detector was activated; however, the VOC and CO sensors had already reached output voltage saturation. The early rise in VOC and CO at T = 6 min suggests a 15 min advantage for early fire detection compared to the smoke detector.

A reference detection threshold was set at 200% of the baseline voltage (1.0 V), meaning 2.0 V was considered a significant alert level, and this level was reached at T = 6 min. As the fire progressed, gas permeability increased in the cavity, and by the end of the experiment, all sensors in this area were rendered inoperative due to high temperatures and wire insulation failure.

A parallel evaluation was conducted for the hollow section with mineral wool insulation (Figure 7b). In this case, the commercial smoke detector was not triggered, and the VOC and CO sensors responded more slowly. The structure burned through between T = 2600 and 2800 s, as indicated by a single thermocouple placed near the flame; other sensors remained below 100 °C. Noise in the smoke sensor signal toward the end was attributed to thermal damage of PVC cable insulation, confirmed during post-experiment inspection.

A notable observation was the differing behavior of gas sensors and commercial smoke detectors. Gas sensors (VOC, CO, CO_2_) detect molecular species, while optical smoke detectors respond primarily to particulate matter. The passage of smoke particles through mineral wool is limited, and potential filtering or de-sublimation effect cannot be excluded.

These results highlight the potential advantage of gas-based fire detection within structural cavities, offering earlier and more reliable fire identification in insulated assemblies such as those presented in Figure 2 and Figure 4. The same color was used for the same sensors (i.e., black = CO_2_), showing comparable results between insulated and uninsulated hollows.

### 6.4. Evaluation of Moisture Detection Systems and IAQ Data

Data from the IAQ and MoistureGuard systems [21] were also processed to complement the overall assessment. These sensors provided additional readings of temperature, humidity, and carbon dioxide (CO_2_), particularly in the hollow and ceiling sections of the structure. The IAQ units, positioned close to the gas sensors, offered valuable insights into CO_2_ buildup during the experiment. With a rated CO_2_ sensing range of up to 20,000 ppm (guaranteed) and an extended but unguaranteed response up to 40,000 ppm, these sensors reached saturation as combustion progressed—validating the high-intensity conditions inside the structure.

Some IAQ units located near the burn-through zones were ultimately destroyed, confirming the severity of thermal exposure. Although full IAQ data, including next VOC, temperature and humidity trends, are not presented here due to space constraints, the CO_2_ curves were used as reference signals to support the interpretation of other sensor data. Their inclusion provided redundancy and strengthened the reliability of fire development insights.

### 6.5. Evaluation of Moisture Detection System Battery and Temperature

In addition to environmental sensing, specific K-type thermocouples were installed within the wireless MoistureGuard units [21] to monitor the internal temperature of their Li-SOCl_2_ (lithium thionyl chloride) batteries. This was carried out to evaluate potential thermal risks posed by the sensor’s battery heating during a fire. While these results are not included in this paper, the data serves as a preliminary assessment of the fire resilience of Li-SOCl2 battery-powered wireless sensing systems in timber buildings, supporting future safety considerations and sensor placement strategies.

### 6.6. Evaluation of Pyrometer Data

Two spot pyrometers (range up to 1100 °C) were used to monitor the experiment from approximately 1 m. One device observed the facade opposite the expected center of the flame burner. The recorded surface temperature rose only slightly—from an initial 18 °C (ambient laboratory temperature) to 24 °C at the end of the 60 min test—indicating no burn-through or significant heat transfer through the facade. This confirmed the suitability of our decision to keep the simplified facade design for the experiment.

The second pyrometer was positioned above the joint between the ceiling and horizontal structure, viewed from the second floor. Here, temperatures remain below 42 °C, with fluctuating values likely influenced by airflow and flue gases from the burner and around the burning specimen. This joint was of particular structural interest. Due to space constraints, detailed pyrometer data are not included in this paper. The pyrometer was thermally insulated from hot air gases and fire.

### 6.7. Evaluation of Smoke Detectors Within the Wall Structure

Commercial smoke detectors (FDR-26-S, optical-smoke principle, certified EN 54 part-7) embedded within the test specimen were assessed qualitatively (Figure 7), based on binary outcomes (detection/no detection). Their behavior is discussed in the relevant sections above, particularly in comparison to gas sensors and thermal data after the experiment detectors were assessed (Figure 5). Note: FDR-26-S (VARIANT plus s r.o., Prague, Czechia)

### 6.8. Evaluation of Gas Sensor Response

Gas sensors (Fermion) based on MEMS [23] and MOS technology were evaluated in correlation with ambient CO_2_ and temperature data. These analog sensors provided a 0–3.3 V output connected to the NI DAQ module. The sensors measured voltage (expecting the proportional reaction [23]), reflecting the uncalibrated concentration of VOC and CO gases. Therefore, they were not converted to ppm or percentage concentrations.

As shown in Figure 8, the CO sensors exhibited a faster response than the VOC and CO_2_ sensors (Figure 7 and Figure 8), which saturated approximately five minutes later. This time-shifted behavior highlights a complementary detection dynamic; combining CO and VOC sensors may improve early fire detection reliability through signal correlation.

## 7. Discussion

A global assessment of the experiment confirms that the uninsulated hollow section experienced complete burn-through, resulting in damage to several sensors and cables. In contrast, the insulated section sustained minimal sensor damage—limited to those positioned closest to the fire source, such as directly above the burner and below the ceiling. The combined analysis of gas sensors and temperature data revealed clear correlations and demonstrated a significant detection advantage over commercial smoke detectors. Early gas detection (CO and VOCs) occurred well before smoke activation, supporting the effectiveness of embedded gas sensors for early warning in timber structures.

From a practical point of view, future experiments could benefit from simplification and focusing on individual structural parts (e.g., walls and ceilings). Furthermore, at the same time, the gas analysis could be improved by using a better performing method (GC or FTIR) and/or with calibrated or at least verified (MEMS, MOS) sensors to obtain more accurate quantitative results. From a structural point of view, it is advantageous to focus on improving the airtightness of the hollow and specimen (keeping airtightness up to structural failure) in order to clearly exclude the possible influence of fumes or CO_2_ originating in another part of the test sample or from the burner.

## 8. Conclusions

Based on the experiment, analysis, and limitations encountered, the following conclusions were drawn:

o  Feasibility of Low-Cost Gas Sensors: Despite limited access to advanced gas analyzers (e.g., FTIR or GC), commercially available gas sensors—though affected by cross-sensitivity—proved effective in detecting fire-related gases (VOC, CO, CO_2_) early in the fire development phase. Early fire detection is possible, but it is necessary to use multiple sensors and such a detection system must be adjusted to the types of materials used in the structure.
o  Early Detection Advantage: Gas sensors demonstrated a significant time advantage (up to +15 min) in detecting fire precursors compared to commercial optical smoke detectors. This early detection window is critical for fire and safety units’ responses (benefits visualized in Figure 1).
o  Use of Redundant Detection Methods: Incorporating temperature sensors within the structure provided an additional layer of verification, improving the reliability of detection (we take into account that the sensitivity of the gas sensors changes over time). This redundancy strengthens the case for hybrid systems combining gas and thermal sensing for early fire warning. Such an early fire detection system should be set to the conditions (precursor gas portfolio) of the specific materials used in the construction of the monitored building part.
o  Limitations of Current Gas Sensor Technologies: The study acknowledges the limitations of semiconductors (MOS and MEMS) and widely used catalytic gas sensors, including gas cross-sensitivity, sensor aging, and calibration drift. These factors introduce uncertainty in gas identification and signal interpretation—which means that calibration is required regularly over long term periods (1, 2, 5 years) while redundant detection methods can minimalize false alarms and gas analysis can be confirmed by other factors (i.e., temperature, another gas precursor).
o  Need for Sensor Calibration and Maintenance: Prior to future experiments or field deployment, gas sensors (especially CO, CO_2_ and VOC) should be calibrated or verified to ensure their accuracy for carrying out the experiment. Furthermore, for commercial use, the sensors should be installed in a way that allows easy replacement or recalibration over time. For VOCs, a representative gas or organic compound should be determined to which the sensor will be calibrated, for example, the gas from Table 1.
o  Improvement in Experimental Methodology: While a gas bag sampling with Tedlar® was considered, potential sample degradation and handling constraints made it unfeasible. FTIR with a heated gas sampling tube remains a recommended method for more precise gas analysis in future studies. GC is less practical for our current fire lab premises.
o  Practical Implications for Timber Buildings: Given the anticipated evolution of legislation regarding multi-story timber buildings in the Czech Republic, and the widespread use of wood-based structures in other EU countries, the development of reliable in-structure fire detection systems is timely and relevant. Just as a moisture monitoring system inside the structural components of wooden buildings has found its application, this early fire detection system can also bring benefits to building users and owners.
o  This study holds significant value in advancing fire safety innovation for timber buildings, particularly as building regulations increasingly support multi-story timber construction. It demonstrates that embedded sensor systems have the potential to outperform traditional smoke detectors by enabling earlier fire detection through gas and thermal sensing. The research lays the foundation for developing hybrid detection systems that enhance reliability and response time. Importantly, it validates it as a practical and scalable approach using commercially available technology suitable for real-world applications. Beyond its technical contributions, the study offers actionable insights for architects, engineers, and policymakers working toward safer, smarter, and more sustainable building practices.

## Figures and Tables

**Table 1:** Ignition temperatures and gases released from construction materials used in simplified spruce timber construction.

Construction Material	Ignition Temperature (°C)	Ignition Time (min)	Pre-Ignition Gases	Post-Ignition Gases	Quantities (Typical Ranges)
OSB	300	1.8	Formaldehyde, Acetic acid, CO, CO_2_, Isocyanates, Ammonia	CO_2_, CO, HCN, Formaldehyde, Acrolein, Benzene, NOx, PAHs	CO: ~50–100 g/kg CO_2_: ~200–300 g/kg HCN: ~1–5 g/kg Formaldehyde: ~1–3 g/kg
Plaster board	1450	does not burn	Water vapor, Trace CO, Trace VOCs from paper	CO_2_, CO, Formaldehyde, Acrolein (from paper), Water vapor	Water vapor: ~1.7 kg/m^2^ CO_2_: ~0.1 kg/m^2^ CO: trace (ppm)
Humid membrane	330–410	0.1	Methane, Ethylene, Formaldehyde, CO Acetaldehyde, Acrolein	CO_2_, CO, PAHs Formaldehyde, Acrolein, Benzene, Soot	CO: ~100–200 mg/g CO_2_: ~500–1000 mg/g Acrolein: ~350 mg/g Formaldehyde: ~2–3 mg/g
Mineral wool	>1000	does not burn	Formaldehyde, Phenol, CO, CO_2_, Ammonia, HCN (trace)	CO_2_, CO, HCN, Isocyanates (trace), Water vapor	CO: trace–tens of ppm, CO_2_: low, HCN: trace ppm, Formaldehyde: trace ppm

Current commonly used commercial smoke (fire) detector technologies are based on the previously described three principles. These sensors are commonly used, and their testing and parameters are subject to standards such as EN 54-5 [12], EN 54-7 [13] and EN 14604 [14] for standalone units [15].

**Table 2:** Measurement systems used on test sample.

Measurement Subsystem	Sensors Used	Purpose
Fire laboratory control system (“FLCS”)	Thermocouples Gas analysis in exhaust hood	Measures temperatures on test specimen and on burner. Experiment control purposes.
PC with NI data acquisition (“DAQ”) unit	Fermion gas sensors Smoke sensors	Gas (MOS) sensors and smoke sensors to monitor CO, VOC, and smoke in specimen. Used to compare these sensors.
PC with USB interface	Pyrometers	Observing specimen temperatures from distance.
Moisture sensors with integrated temp/humidity	Wired and Wireless timber moisture sensors including temp./humidity sensing.	Observing specific points for moisture, temperature, humidity. For robustness.
	data	data
Indoor air quality sensors (“IAQ”)	Ambient air sensors including temp./humidity sensing.	Observing CO_2_, VOC, temperature, and humidity.

**Figure A1:**
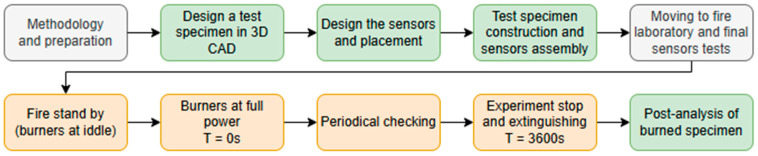
Conduct of experiment in a flow-chart.

**Figure A2:**
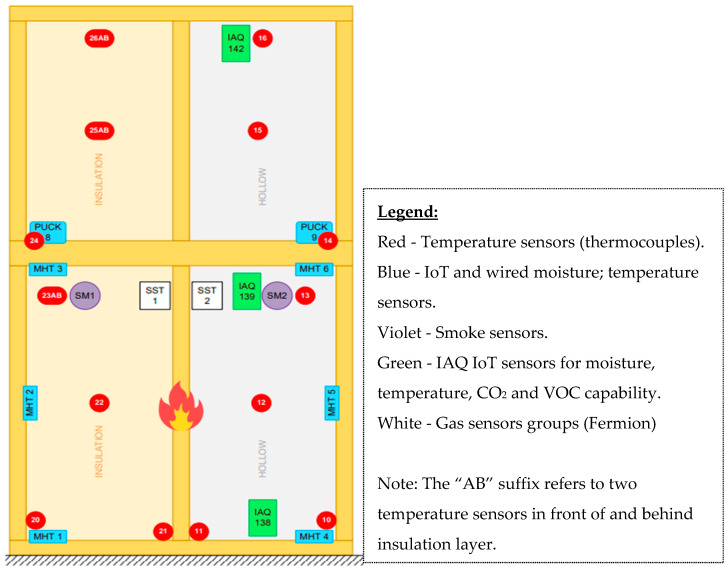
Sensor placement.

## Data Availability

Data are available on request from the authors.

## Appendix A

The Appendix contains attachments that will help you understand the design of the experiment.

### Appendix A.1. Experiment Flow Diagram

This flow diagram describes the experiment in medium detail (Figure A1). This entire phase took several months, as it depended on the workload of other departments and collaboration.

### Appendix A.2. Sensors Placement on Test Specimen

Figure A2 describes placement sensors on test specimen construction. The figure describes the location of sensors when viewed from inside the exposed structure, excluding the ceiling.

## References

[1] Statistical Yearbooks—2024 (FRS Czech). https://hzscr.gov.cz/clanek/statisticke-rocenky-hasicskeho-zachranneho-sboru-cr.aspx.

[2] Khan F., Xu Z., Sun J., Khan F.M., Ahmed A., Zhao Y. (2022). Recent Advances in Sensors for Fire Detection. Sensors.

[3] Gu H., Wang Z., Hu Y. (2012). Hydrogen Gas Sensors Based on Semiconductor Oxide Nanostructures. Sensors.

[4] Challands N. (2010). The relationships between fire service response time and fire outcomes. Fire Technol..

[5] Ramachandran G. (1986). Exponential Model of Fire Growth. Fire Saf. Sci..

[6] Hakkarainen T. (2002). Post-flashover fires in light and heavy timber construction compartments. J. Fire Sci..

[7] Nejzajímavější České Dřevostavby—Remspace. https://www.remspace.cz/clanek/nejzajimavejsi-ceske-drevostavby/.

[8] Konstrukce Dřevostaveb. https://www.rdrymarov.cz/schemata-sten-a-stropu.

[9] Nocquet T., Dupont C., Commandre J.-M., Grateau M., Thiery S., Salvador S. (2014). Volatile species release during torrefaction of wood and its macromolecular constituents: Part 1—Experimental study. Energy.

[10] Kinoshita H., Türkan H., Vucinic S., Naqvi S., Bedair R., Rezaee R., Tsatsakis A. (2020). Carbon monoxide poisoning. Toxicol. Rep..

[11] (2021). Rockwool Safety Data Sheet for Stone Wool. https://www.rockwool.com.

[12] (2018). Fire Detection and Fire Alarm Systems—Part 5: Heat Detectors—Point Heat Detectors.

[13] (2018). Fire Detection and Fire Alarm Systems—Part 7: Smoke Detectors—Point Smoke Detectors that Operate Using Scattered Light, Transmitted Light or Ionization.

[14] (2005). Smoke Alarm Devices.

[15] (2022). Standard Test Methods for Fire Tests of Building Construction Materials.

[16] (2025). Fire-Resistance Tests—Elements of Building Construction—Part 1: General Requirements.

[17] Liang Z., Lin S., Huang X. (2023). Smoldering ignition and emission dynamics of wood under low irradiation. Fire Mater..

[18] Drysdale D. (2011). An Introduction to Fire Dynamics.

[19] (2024). Fire Classification of Construction Products and Building Elements—Part 2.

[20] (2010). Reaction to Fire Tests for Building Products—Conditioning Procedures and General Rules for Selection of Substrates.

[21] Senzomatic (About System). https://senzomatic.com/en/about-us/.

[22] Jan Vcelak Indoor Air Quality Sensors. https://www.uceeb.cz/en/iaq-indoor-air-quality-sensors/.

[23] SGS Sensortech Technical Datasheet (MICS-5524). https://www.sgxsensortech.com/content/uploads/2014/07/1084_Datasheet-MiCS-5524-rev-8.pdf.

